# Sour Stomach

**Published:** 1883-06

**Authors:** 


### SOUR STOMACH.

THE stomach is provided by nature with just enough "gastric juice" to dissolve the food that the system requires. Whatever is eaten beyond what is needed, has no gastric juice to dissolve it, and being kept at the temperature of the stomach, which is about 100 degrees, it begins to ferment in a few hours, just as new cider turns sour when kept warm for a short time. In the process of souring, gas is generated as in the cider-barrel, the bung is thrown out because of the expansion of the liquid. So with the stomach. It may be partially filled by a meal; but if more has been swallowed than wise nature has provided gastric juice for, it begins to sour, to ferment, to distend, and the man feels uncomfortably full. He gets some relief by belching up gas, but as the fermentation goes on he gets colic, or some other discomfort, which lasts for several hours, when nature succeeds in getting rid of the surplus, and the machinery runs smoothly again. But if these things are frequently repeated the machinery fails to rectify itself, loses the power of readjustment, works with a clog, and the man is a miserable dyspeptic for the remainder of life ; and all from his not having wit enough to know when he had eaten enough, and being foolish enough, when he had felt the ill effect of thus eating too much, to repeat the process an indefinite number of times, and all for the trifling object of feeling good for the brief period of its passing down the throat. For each minute of that good he pays the penalty of a month of suffering such as only a dyspeptic can appreciate.

When the dyspepsia is in the stomach there is belching or eructations, when in the bowels, the gases pass downward, chronic diarrhœa sets in, with its relentless wasting away of flesh and strength, and finally of life itself; or fearful neuralgias rack the whole frame, first in one place, then in another, making life a burden instead of a blessing.

When there is habitual acidity or wind after meals, it is the result of an error in eating ; oftener of quantity than quality; and common sense dictates taking less and less each meal, and adhering to the amount which can be taken without any subsequent discomfort whatever.

---

Burns and Scalds are instantly relieved by immersing the parts in cold water; then in severe cases, send for the doctor, and while he is coming, cover the injured parts half an inch deep with common dry flour; keep the bowels acting daily, and take nothing but gruel, soup, stale bread and baked fruits. This is the safest, best, speediest and most certain cure ever made known for burns.

